# Clinical Pathways Based on Integrative Medicine in Chinese Hospitals Improve Treatment Outcomes for Patients with Acute Myocardial Infarction: A Multicentre, Nonrandomized Historically Controlled Trial

**DOI:** 10.1155/2012/821641

**Published:** 2012-09-13

**Authors:** Lei Wang, Minzhou Zhang, Liheng Guo, Jianyong Qi, Haiming Luo, Hankang He, Xiaolong Wang, Haiyu Yang, Yang Wu, Canming Miu, Xiaohu Chen, Jiashin Wu

**Affiliations:** ^1^Intensive Care Unit (ICU), Guangdong Provincial Hospital of Traditional Chinese Medicine, Guangzhou 510120, China; ^2^Intensive Care Unit (ICU), 2nd Affiliated Hospital of Guangzhou University of Traditional Chinese Medicine, Guangzhou 510120, China; ^3^Yueyang Hospital of Integrated Chinese and Western Medicine, Shanghai University of Traditional Chinese Medicine, Shanghai 200437, China; ^4^3rd Affiliated Hospital, Guangxi College Traditional Chinese Medicine, Guangxi 545001, China; ^5^Shuguang Hospital, Shanghai University of Traditional Chinese Medicine, Shanghai 200021, China; ^6^Wuyi Hospital of Traditional Chinese Medicine, Jiangmen, Guangdong 200021, China; ^7^Oriental Hospital, Beijing University of Traditional Chinese Medicine, Beijing 100078, China; ^8^Zhongshan Hospital of Traditional Chinese Medicine, Zhongshan, Guangdong 528400, China; ^9^Jiangsu Provincial Hospital of Traditional Chinese Medicine, Nanjing, Jiangsu 210029, China; ^10^Morsani College of Medicine, University of South Florida, Tampa, FL 33612, USA

## Abstract

*Objective*. To determine the impact of an integrative medicine clinical pathways (CPs) on the length of in-hospital stay and on outcomes for patients with acute myocardial infarction (AMI). *Methods*. A multicenter nonrandomized controlled trial enrolling 197 consecutive patients with AMI at eight urban TCM hospitals was conducted between 1 January 2010 and 31 October 2010. These patients were enrolled in the interventional group after the CPs had been implemented. The control group included 405 patients with AMI from eight hospitals; these patients were treated between 1 January 2008 and 31 December 2009, before the CPs were implemented. Outcome measures were the length of hospital stay costs of medical care, and major cardiovascular events (MACEs) during hospitalization. *Results*. Compared with the control group, the patients in intervention group had a shorter length of hospital stay (9.2 ± 4.2 days versus 12.7 ± 8.6 days, *P* < 0.05), and reduced healthcare costs in hospital (46365.7 ± 18266.9 versus 52866.0 ± 35404.4, *P* < 0.05). There were statistically significant differences in MACE between the two groups during the hospitalization period (2.5% versus 6.9%, *P* = 0.03). *Conclusion*. These data suggest that the development and implementation of the clinical pathways based in Integrative Medicine could further improve quality of care and outcome for patients with AMI.

## 1. Introduction

Acute myocardial infarction (AMI) is a serious cardiovascular disease and is a leading cause of death worldwide. In recent years, the AMI incidence and mortality has decreased significantly in America because early reperfusion and drug treatment has been standardized [1]. However, it is estimated that with further economic development, aging of the population, and changes in diet and physical activity in China, the absolute number of AMI events and deaths will increase dramatically in the next two decades [2]. There is also a discrepancy between guideline recommendations and the current AMI management in most Chinese hospitals [3]. Therefore, strong evidence-based initiatives to improve patient management will be critical to address this challenge.

TCM has been practiced for thousands of years, and it has made great contributions to peoples' health and wellbeing. Epidemiological data has suggested that Chinese herbal preparations may be beneficial in reducing the mortality from AMI, and TCM treatment was shown to help improve the quality of life for AMI patients [4]. TCM hospitals have the ability to perform reperfusion and to use drugs appropriately, and they are also making progress in their effort to follow the Clinical Guidelines. However, there are still situations that arise when using TCM to treat patients with AMI, including a lack of standardized TCM syndrome diagnosis, the need for syndrome differentiation and treatment standardization, and clinical skills in reperfusion and standardized drug treatment, which require further improvement [5].

Clinical pathways (CPs), also known as critical pathways, are management plans that display goals for patients and provide the sequence and timing of actions necessary to achieve these goals with optimal efficiency. As competition in the healthcare industry has increased, CPs have been widely implemented as a method to reduce variation in care and potentially improve healthcare quality. Cardiovascular medicine in particular is an area in which CPs have been used extensively [6]. The evidence-based Integrative Medicine CPs, developed to improve the quality of healthcare, was based on published guidelines and the best research evidence from TCM and Western medicine [7]. Previous research has suggested that CPs may help to reduce costs while improving the quality of care for AMI patients [8]. However, the effectiveness of Integrative Medicine CPs on improving AMI management is unclear.

In previous studies, we developed CPs based on standardized therapy for AMI Integrative Medicine. The standardized management included thrombolysis therapy, primary percutaneous coronary intervention (PCI), antiplatelet and anti-ischemic therapy, and TCM therapy (such as Astragalus injection and compound Danshen dripping pills) to benefit Qi and to activate blood. A small single-center trial suggested that the CPs could reduce the length of the hospital stay and in-hospital health care costs for patients with AMI who underwent PCI [9]. The purpose of this study is to assess further the influence of the Integrative Medicine CPs on care quality and outcomes among AMI patients in TCM hospitals, in a multicenter nonrandomized controlled trial.

## 2. Methods

### 2.1. Study Design and Setting

**Figure 1:**
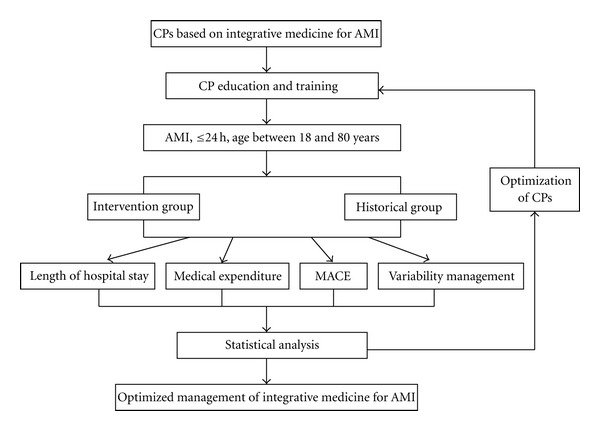
Evaluation of clinical pathways based on integrative medicine for AMI. CPs: clinical pathways, MACE: major adverse cardiac events, AMI: acute myocardial infarction.

 This trial is a multicenter, nonrandomized retrospective study in eight hospitals (Guangdong Provincial Hospital of TCM; Shuguang Hospital of Shanghai University of TCM; Yueyang Hospital of Integrated Medicine of Shanghai University of TCM; Oriental Hospital of Beijing University of TCM; Jiangsu Provincial Hospital of TCM; 3rd Affiliated Hospital of Guangxi College of TCM; Wuyi Hospital of TCM of Jiangmen city; Zhongshan Hospital of TCM) (Figure 1). This study (2008GL-35) was approved by the Ethical Committee of Guangdong Provincial Hospital of Chinese Medicine.

### 2.2. Study Patients

 Inclusion criteria for this study included patients with acute myocardial infarction (onset of chest pain ≤24 h) admitted to emergency, and ages ranging from 18 to 80 years old who agreed to emergency reperfusion therapy (including intravenous thrombolysis or PCI). Exclusion criteria included serious mechanical complications (such as left ventricular free wall rupture, ventricular septal perforation, papillary muscles, and adjacent chordal rupture), concomitant diseases with aortic dissection, acute pulmonary embolism, severe liver failure, renal failure, mental illness, malignancy, hematopoietic tumor, nervous system primary diseases, and pregnancy or lactation [10]. 

In our preliminary study, we found that Qi deficiency and blood stasis were the main TCM syndromes for AMI [11]. In order to implement the AMI CPs conveniently in all hospitals, we considered Qi deficiency and blood stasis as the basic syndrome occasionally accompanied with Phlegm, Yin-deficiency or Yang-deficiency of a single TCM syndrome element. The diagnostic criteria of the Qi deficiency and blood stasis were based on the TCM standard of coronary heart disease, which was formulated by the Cardiovascular Society of the National Association of Integrative Medicine [12].

Sample size was calculated using PEMS 3.1 for Windows software (Sichuan University, Chengdu). The length of in-hospital stay was considered to be one of the most important factors in the calculation of sample size. The standard deviation (SD) of the length of in-hospital stay for the conventional treatment group was 6 days [13], and it was considered clinically significant when the length of in-hospital stay was reduced by 4 days. If *α* = 0.05, power = 0.90, and *β* = 0.10, the estimated total sample size is 256 patients. Taking into account a 15% dropout rate, the total sample size is 294 patients.

In the study protocol, the planned sample size was 240 consecutive patients who were enrolled into the intervention groups after pathway implementation. There were 450 consecutive patients, admitted to the eight hospitals between 1 January 2008 and 31 December 2009, prior to CP implementation, who were included as a historical control group. The Guangdong Provincial Hospital of TCM planned to enroll 100 patients for the intervention group and 100 patients for the control group. Additionally, each of the other 7 hospitals planned to enroll 20 patients for the intervention group and 50 patients for the control group.

### 2.3. Intervention

The patients in the historical control group received conventional management determined by a physician, which included Western medicine and nonstandardized TCM therapy. The patients in the intervention group were treated according to the standardized management plan as determined by the CPs. The Western medical treatment consisted of reperfusion therapy and aspirin, clopidogrel, low molecular weight heparin (LMWH), *β* receptor blocker, and angiotensin-converting enzyme inhibitors (or angiotensin II receptor blocker), according to the 2007 updated guidelines for the management of patients with ST-segment elevation myocardial infarction (STEMI) [14]. 

For Qi deficiency and blood stasis, the standard TCM technique performed in the intervention group was 30 mL Astragalus injection (Astragalus, Zhengda Qingchunbao pharmaceutical company) mixed with 250 mL 5% glucose, which was infused intravenously once per day, and 10 particles of compound Danshen dripping pills (Salvia, Pseudo-ginseng, Borneol, Tasly Group) 3 times a day. Instead of Astragalus injection, Gualou Xiebai Banxia Tang (Trichosanthes 15 g, Bulbus allii macrostemonis 20 g, Pinelliae 15 g) was administered to patients with cold-phlegm syndrome, Wen Dan Tang (Poria 15 g, Dried tangerine peel 10 g, Pinellia 15 g, Caulis bambusae in taenia 15 g, Fructus aurantii 15 g) was administered for patients with heat-phlegm syndrome, 30 mL Shen Mai injection (Ginseng, Radix, Zhengda Qingchunbao pharmaceutical company) mixed with 5% glucose injection infusion was administered for Yin-deficiency, and 30 mL Shenfu (tuber, red ginseng, Sanjiu Ya pharmaceutical company) in 5% glucose intravenous infusion was administered for Yang-deficiency. All the treatments were administered for 1 week. 

### 2.4. Outcome Measures

 The primary outcome was the length of the in-hospital stay. Discharge standard, for patients to be discharged from the hospital with stable life signs (hemodynamic, electrocardiogram, and cardiac function) and without the symptoms of myocardial ischemia, was determined according to “Clinical pathways of ST-segment elevation myocardial infarction” (2009 version) by the Medical Administration of the Ministry of Health [15]. The secondary outcome was the major cardiovascular events (MACE) and economic evaluation during the period of hospitalization. MACE is defined as death, nonfatal myocardial reinfarction, stent thrombosis or target vessel revascularization. Total medical costs include treatment costs, operation costs, drug costs, nursing costs, inspection fees, and bed charges. All data were analyzed using SPSS 17.0 (IBM Corporation, Armonk). Measurement data were presented as mean ± ST. Count data were presented as the frequency and constituent ratio, and analyzed using the chi-square test or the exact test exact probability method. For measurement data, two samples were compared using the Mann-Whitney *U* test. The statistical tests used were two-sided tests, and *P* < 0.05 was considered a statistically significant difference. 

## 3. Results

Between 1 January 2010 and 31 October 2010, a total of 250 consecutive patients fulfilling the inclusion criteria were initially evaluated as the intervention group, and 53 patients were excluded because of severe mechanical complications or severe liver failure and renal failure. A total of 450 patients admitted to eight hospitals from 1January 2008 to 31 December 2009 were screened for the historical control group, and 45 patients were excluded because of severe mechanical complications or concomitant diseases. As a result, there were 197 patients enrolled into the intervention group and 405 patients enrolled into the historical control group.

### 3.1. Clinical Features in the Intervention and Historical Control Groups

Of the 602 patients, 514 (85%) were admitted for ST-segment elevation myocardial infarction (STEMI) and 71 (15%) for non-ST-segment elevation myocardial infarction (NSTEMI). There were 567 patients (94%) who underwent emergency PCI and 35 patients (6%) who received intravenous thrombolysis. Patient characteristics were not significantly different between the intervention and control groups. The only exceptions were a slightly increased number of patients with a family history of coronary disease in the control group compared to the intervention group, and small increase in the number of patients in the intervention group who had hyperlipidemia or who currently smoked, compared to the control group (Table 1).

### 3.2. Drug Treatments

Comparing the main Western medicine drug treatments in both groups, there were no statistically significantly differences among antiplatelet therapy (aspirin, clopidogrel), beta receptor blockers, ACEI (or ARB), and nitrate (*P* > 0.05). However, the prescribing frequency of low molecular weight heparin (LMWH) and statins in the intervention group was higher in the intervention group than in control group (*P* < 0.05; Table 2).

The rate of Chinese medicine decoction use in the treatment group was significantly higher than in the control group (*P* < 0.01). There were also significant differences among Compound Danshen dripping pills, Qi-benefitting agents, and blood-activating agents (*P* < 0.05). The frequency of injections benefitting Qi was higher in the treatment group than in the control group, whereas injections promoting blood circulation had a lower frequency in the treatment group compared to the control group (Table 3).

### 3.3. The Average Length of In-Hospital Stay

The length of in-hospital stay showed a skewed distribution in both groups, and therefore a nonparametric test was used. The average length of stay in the treatment group was 3.5 days less than that of the control group, which was statistically significant (*P* < 0.01; Table 4).

### 3.4. The Total In-Hospital Costs

The average hospitalization costs had a skewed distribution in both groups, so a nonparametric test was used. In the intervention group, the average total in-hospital charges were ¥48047.3 when it was adjusted by the price index, which reduced to ¥4820.00 compared with the control group. There were statistically significant differences between intervention groups and control groups. (*P* < 0.01; Table 5).

### 3.5. The Incidence of Major Cardiovascular Events (MACEs)

The major adverse events of death, nonfatal myocardial reinfarction, stent thrombosis, and target vessel revascularization (TVR) occurred in 2.5% of patients (5 of 197) in the interventional group and in 6.9% (28 of 405) of those in the control group during hospitalization (*P* = 0.03; Table 6). Three patients in the intervention group died due to cardiac shock, and 22 patients died in the historical control group due to cardiac shock (10 patients), severe heart failure (4 patients), ventricular fibrillation (5 patients), and multiple organ dysfunction syndrome (3 patients). The majority of MACE incidents were death during hospitalization (1.5% versus 5.4%, *P* = 0.03). There was no statistical difference in nonfatal myocardial reinfarction, stent thrombosis, and TVR between two groups (Table 6).

## 4. Discussion

The number of patients in China who develop and present to hospitals with acute coronary syndrome will increase in the near future. China's fee-for-service payment system has resulted in a rapid cost increase, inefficiencies, poor quality, unaffordable health care, and an erosion of medical ethics [16], and improvement of patient management, together with health system reform, is urgently required. CPs are management plans that specify goals for patients and provide the sequence and timing of the actions necessary to achieve these goals with optimal efficiency. Several areas for improving patient care using CPs have been identified, including increasing the use of guideline-recommended medications, reducing variation in care, targeting the use of cardiac procedures, and reducing the length of the hospital stay [17]. Therefore, the Ministry of Health in China has encouraged the implementation of CPs in cardiovascular disease to control medical cost and to improve healthcare quality.

To standardize the AMI treatment-based integrative medicine in TCM hospitals, we conducted this study and evaluated the efficacy of CPs in eight TCM hospitals. In our study, there were more patients with hyperlipidemia or who were current smokers, and fewer patients with a family history of coronary disease in the intervention group than in the historical control group. These data indicate that, with diet and life-style changes in China, smoking and hyperlipidemia have emerged as key risk factors leading to AMI, especially for younger people.

### 4.1. Length of In-Hospital Stay

A previous epidemiological study showed that the average length of hospital stay was from 11.6 to 13.7 days in China for the patients with AMI who were admitted to hospital within 12 h after onset of chest pain [13]. Our trial indicated that the average length of stay in the treatment group (after implementation of CPs) was 3.5 days less than the control group (9.2 ± 4.2 days versus 12.7 ± 8.6 days, *P* < 0.01). The length of the hospital stay after AMI depends on many factors, including department policies, insurance coverage, age, and patients' complications. However, the length of the hospital stay depended more on the damaged myocardium duration of recovery because there were few changes in the medical policy and insurance coverage from 2008 to 2010, and there are no statistical differences in age, cardiac function, and other clinical characteristics between the interventional and historical groups. In this study, we consistently followed the hospital discharge standards, which ensured that the patients would be discharged from hospitals in a stable condition. Therefore, the reduction in the length of stay observed in this study is attributed to the potential improvement of healthcare quality and the decreased variation in care conferred by the use of CPs.

### 4.2. Total In-Hospital Medical Costs

China's current strategy to improve payment for health services has made some positive changes; however, the rapid increase in health expenditure and inappropriate treatment concerning individuals and governments resulting from China's fee-for-service payment and a price schedule that overpays for drugs and high-technological diagnostics tests has led providers to overprescribe drugs and diagnostic tests [18]. Control of the high out-of-pocket healthcare payments when patients have inadequate insurance coverage in many parts of China is critical. Our study showed that, after the price index adjustment, the total medical expenditure during hospitalization in the intervention group was reduced compared to the historical control group (¥46365.70 versus ¥52866.00, resp., *P* < 0.01). This suggested that the CPs based on integrative medicine could decrease the cost of hospitalization through a reduction in the length of a patient's in-hospital stay and in the potential overprescription of drugs and diagnostic tests.

### 4.3. Health Providers' Compliance with the Guideline

Despite strong evidence for the benefits of AMI patient management using antiplatelet agents, LMWH and lipid-lowering therapies, reports from the CPACS study indicated that physician compliance with guideline recommendations and sustained use of medical therapy remains suboptimal [19]. Statins and LMWH are effective in reducing mortality and serious coronary events in patients with AMI. Despite the increased usage rate of these therapies in TCM hospitals, data has shown a lower prescribing rate in TCM hospitals than in Western Medicine hospitals [20]. Our results demonstrate that the use rates of LMWH (100% versus 89%) or statin lipid-lowering drugs (100% versus 96%) in the intervention group was higher than in the historical control group, respectively, which revealed that CPs have the potential capacity to improve health providers' adherence to published AMI guidelines and to close the gap between the practice and the guideline. However, the prescribing frequency of *β* receptor blockers and ACE inhibitors were not as high as expected in the intervention group (81% and 82%, resp.). These findings are not unique to TCM hospitals; data from CPACS has consistently demonstrated that *β* receptor blockers and ACE inhibitors are not being used as often or as long as they should be, which reflects the physicians' fears that these drugs may lead to dynamitic deterioration in patients with cardiac shock, acute heart failure, or low blood pressure [19].

### 4.4. Management of TCM after Reperfusion Therapy

TCM plays an important role in the current treatment of AMI especially in TCM hospitals. In the past 10 years, blood-activating had emerged as a main TCM method for treating patients with AMI, thus leading to wide prescribing of Chinese medicine intravenous preparations that are clinical blood-activating agents [5]. As a result, there is more usage of blood-activating intravenous agents in the historical group (63%), as shown in Table 3. However, in our previous study that related the distribution and evolvement of syndrome elements during the perireperfusion period, we found that reperfusion treatments play a vital role in activating circulation in the TCM theory, and that Qi deficiency and blood stasis are the main syndromes after reperfusion therapy [11]. An analysis of 5284 patients with coronary artery disease indicated that the top two TCM patterns were blood stasis (79.3%) and Qi deficiency (56.5%) [21]. Therefore, Qibenefitting and blood activating should become the main TCM treatment, replacing blood-activating alone. In this study, Qi-benefitting intravenous agents (e.g., Astragalus, Shen Mai injection and Shenfu injection) and compound Danshen dripping pills were used as the standardized TCM management after reperfusion. Thus, it is reasonable that the rate of use of these agents in intervention group are higher than in the historical group (*P* < 0.01). Other trials indicated that Astragalus injection was effective in reversing left ventricular remodeling and improving left ventricular function in patients with AMI [22], and that Salvia miltiorrhiza extract (the main ingredients of compound Danshen dripping pills) affords protection against isoproterenol-induced myocardial infarction [23], which demonstrated the possible mechanism of accelerating cardiac function recovery using Qi-benefitting and blood-activating compounds. Also, CPs application based on integrative medicine guarantees an increase in the standardized usage of TCM therapy (97% versus 84%).

### 4.5. Major Adverse Cardiac Events

Despite wide implementation of CPs in cardiovascular disease, no controlled study has shown that CPs could reduce the incidence of the death or MACE in patients with AMI. Our research indicated that, compared with the historical group, the incidence of death and MACE during hospitalization was lower in the intervention group compared to the control group (1.5% versus 5.4%, 2.5% versus 6.9%, resp., *P* < 0.05). The reasons for this encouraging outcome are complex. Multiple factors including an increase in prescribing drugs, recommended by AMI guidelines, standardized use of TCM based on Qi-benefitting and blood-activating, and a decrease in the variation in care attributed to an improvement in healthcare.

## 5. Conclusions

Integrative medicine treatment, combining TCM and conventional medicine, has been the most representative characteristic for patients with coronary heart disease in China, especially those in TCM hospitals. We found that, in the current era of published treatment guidelines, implementation of the CPs based on the standardized therapies of integrative medicine could further improve guideline compliance and overall quality of care by reducing the length of stay and medical cost for patients with AMI in Chinese hospitals.

## 6. Limitations of This Study

Although our study revealed the potential improvements in patient outcome by the development and implementation of CPs for AMI patients in China, there are several limitations of the study. First, the duration of the study period was short because of budget limitations, which leaves uncertainty in the long-term outcome of patients with AMI. Second, this study used a nonrandomized retrospective trial design, which may not fully reflect the improvement of CPs on the quality of health care due to potential changes in insurance coverage or policy. Therefore, multicenter large-scale randomized studies are needed to assess prospectively the differential effects of CPs based on integrative medicine versus CPs only based on western medicine. 

## Figures and Tables

**Table 1:** Demographic and clinical features of patients.

Variable	Intervention group (*n* = 197)	Historical control group (*n* = 405)	*χ* ^2^ (Z)	*P* value
Male gender	149 (75.6)	308 (76.0)	0.01	0.91
Age (yrs)	63.42 ± 11.87	63.89 ± 13.20	−0.49	0.63
Hypertension	109 (55.3)	214 (52.8)	0.33	0.57
Diabetes	37 (18.8)	55 (13.6)	2.77	0.10
Hyperlipidemia	58 (29.4)	65 (16.0)	5.88	0.02
Previous coronary disease	14 (7.1)	22 (5.4)	0.66	0.42
Previous stroke	20 (10.2)	34 (8.4)	0.01	0.93
Current smoker	106 (53.8)	196 (48.4)	4.96	0.03
Family history of coronary disease	16 (8.1)	65 (16.0)	10.13	0.00
Clinical pattern				
STEMI	176 (89.3)	338 (87.1)	0.61	0.44
NSTEMI	21 (10.7)	50 (12.9)		
Cardiac function (Killips classification)				
Level I	99 (64.7)	275 (69.1)	−0.96	0.34
Level II	31 (20.3)	70 (17.6)		
Level III	10 (6.5)	23 (5.8)		
Level IV	13 (8.5)	30 (7.5)		
Intravenous thrombolysis	8 (4.1)	27 (6.7)	1.64	0.20
Emergency PCI	189 (95.9)	378 (93.3)		
Vascular lesions				
Single	64 (34.0)	102 (30.0)	−0.37	0.72
Two branch	56 (29.8)	116 (34.1)		
Three branch	65 (34.6)	122 (35.9)		
Stenosis < 50%	3 (1.6)	0 (0.0)		
Stent implantation	1.19 ± 0.63	1.25 ± 0.90	−0.11	0.92

Values are given as number of patients (%) or mean ± SD.

STEMI: ST-segment elevation myocardial infarction; NSTEMI: non-ST-segment elevation myocardial infarction.

PCI: percutaneous coronary intervention.

**Table 2:** Western medicine prescribing frequency.

Variable	Intervention group (*n* = 197)	Historical control group (*n* = 405)	*χ* ^2^	*P*
Antiplatelet	197 (100)	402 (99)	—	1.00*
Low molecular weight heparin	197 (100)	362 (89)	—	0.00*
Statins	197 (100)	388 (96)	—	0.01*
*β* receptor blockers	160 (81)	320 (79)	0.12	0.72
ACE inhibitors (or ARB)	163 (82)	324 (80)	0.64	0.42
Nitrate esters	138 (70)	276 (68)	0.07	0.79
Antiarrhythmic drugs	65 (33)	79 (20)	12.41	0.00

Values are given as number of patients (%).

*Using the exact probability method.

ACE: angiotensin-converting enzyme; ARB: angiotensin II receptor blocker.

**Table 3:** Chinese medicine prescribing frequency.

Variable	Intervention group (*n* = 197)	Historical control group (*n* = 405)	*χ* ^2^	*P*
Chinese medicine therapy	192 (97)	342 (84)	21.93	0.00
Compound Danshen dripping pills	124 (63)	9 (7)	283.91	0.00
Qi-benefitting intravenous agents^*▵*^	153 (78)	176 (43)	62.58	0.00
Blood-activating intravenous agents^*◊*^	0 (0)	256 (63)	—	0.00*

^∗^Using the exact probability method.

^*▵*^Qi-benefitting intravenous agents are TCM injections whose indications are to improve rehabilitation by benefitting Qi, and they include Astragalus injection, Shen Mai injection, and Shenfu injection.

^*◊*^Blood-activating intravenous agents are TCM injections whose indications are to promote rehabilitation by promoting blood circulation and eliminating blood stasis, and they include Salvia injection, Safflower injection, and Tetramethylpyrazine injection et al.

**Table 4:** Length of in-hospital stay.

Variable	Group	*N*	$\overset{-}{x}\pm s$	*M*	*Z*	*P*
Length of stay (days)	Intervention	197	9.2 ± 4.2	9	5.08	<0.001
	Historical	405	12.7 ± 8.6	11		

**Table 5:** In-hospital medical costs.

Variable	Group	*N*	$\overset{-}{x}\pm s$	*M*	*Z*	*P*
Total charges (¥)	Intervention	197	48047.3 ± 18929.4	44198.7	1.83	0.067
	Control	405	52866.0 ± 35404.4	46157.8		
Total charges adjusted by price index (¥)	Intervention	197	46365.7 ± 18266.9	42651.7	2.94	0.003
	Control	405	52866.0 ± 35404.4	46157.8		

**Table 6:** Individual and combined outcome measure of MACE occurrence during hospitalization.

Variable	Intervention group	Historical group	*P*
Death	3 (1.5)	22 (5.4)	0.03*
Nonfatal MI	1 (0.5)	1 (0.2)	0.55*
Stent thrombosis	1 (0.5)	2 (0.5)	1.0*
TVR	0 (0)	3 (0.7)	0.56*

Total	5 (2.5)	28 (6.9)	0.03

^∗^Using the exact probability method; MI: myocardial reinfarction; TVR: target vessel revascularization.
